# Magnetic DNA random access memory with nanopore readouts and exponentially-scaled combinatorial addressing

**DOI:** 10.1038/s41598-023-29575-z

**Published:** 2023-05-25

**Authors:** Billy Lau, Shubham Chandak, Sharmili Roy, Kedar Tatwawadi, Mary Wootters, Tsachy Weissman, Hanlee P. Ji

**Affiliations:** 1 Division of Oncology, Department of Medicine, Stanford University School of Medicine, Stanford, CA 94305 USA; 2 Stanford Genome Technology Center, Stanford University, Palo Alto, CA 94304 USA; 3 Department of Electrical Engineering, Stanford University, Stanford, CA 94305 USA

**Keywords:** Biotechnology, DNA sequencing, Bioinformatics

## Abstract

The storage of data in DNA typically involves encoding and synthesizing data into short oligonucleotides, followed by reading with a sequencing instrument. Major challenges include the molecular consumption of synthesized DNA, basecalling errors, and limitations with scaling up read operations for individual data elements. Addressing these challenges, we describe a DNA storage system called MDRAM (Magnetic DNA-based Random Access Memory) that enables repetitive and efficient readouts of targeted files with nanopore-based sequencing. By conjugating synthesized DNA to magnetic agarose beads, we enabled repeated data readouts while preserving the original DNA analyte and maintaining data readout quality. MDRAM utilizes an efficient convolutional coding scheme that leverages soft information in raw nanopore sequencing signals to achieve information reading costs comparable to Illumina sequencing despite higher error rates. Finally, we demonstrate a proof-of-concept DNA-based proto-filesystem that enables an exponentially-scalable data address space using only small numbers of targeting primers for assembly and readout.

## Introduction

DNA has properties that allow it to store and maintain genetic information for extended periods of time. As a result, DNA provides a potential solution to the need for massive data storage. DNA molecules provide extremely high data density and long-term durability^1,2^. In comparison to current magnetic and solid-state data storage technologies, DNA has a potentially higher storage density in addition to natural redundancy from molecular copying. Magnetic and solid-state storage technologies can be stably maintained up to decades but undergo progressive media degradation. In contrast, under optimal storage conditions, the physical stability of DNA is on the order of thousands of years^1,2^. There is ongoing work to develop DNA storage technologies with data writing into encoded oligonucleotides and reading through next generation sequencers^3^.

Data storage systems revolve around the concept of random access of data elements from a larger stored pool of multiple data stores or ‘files’. New molecular technologies will be required for recovering stored data in synthetic DNA on an arbitrarily large scale with random access features. Earlier studies used wholesale sequencing of the entire archive followed by bioinformatic extraction of small parts of data from the dataset^4,5^. Recently, some groups have used conventional PCR to amplify the target files of interest for sequence-based reading^6–8^. However, this method has limitations on its scalability to the large numbers of DNA-based data files. For DNA-based data, reading files typically requires PCR amplification with individual pairs of targeting primer oligonucleotides. Accessing these files also leads to consumption and potential representative distortions of the DNA molecular information. These issues are a direct result of the molecular preparation and amplification for the sequencing process. Recent work has shown it is possible to re-amplify DNA oligonucleotide pools^9^ but an ideal solution would be potentially lossless reading of a file from the source DNA.

Errors in DNA synthesis, artifacts in sequencing, and sequence dropouts also occur frequently. As a result, reading DNA data requires error correction techniques to enable reliable recovery of data and to optimize the coding density and sequencing coverage. Erlich and Zielinski focused on Illumina sequencing with a Fountain code scheme^9,10^. Organick et al. presented a scheme for both Illumina and nanopore sequencing using a variety of methods including multiple sequence alignment (MSA) and Reed Solomon codes^11^ to handle sequence erasure and sequencing errors^7^. Lopez et al. improved the MSA algorithm leading to significantly fewer reads being required for successful decoding with nanopore sequencing^12^. Several theoretical studies focused on various aspects of the DNA-based storage problem such as the information-theoretic capacity in the asymptotic setting^13^, the optimality of various techniques to recover the order of the oligonucleotides^14^, development of indel correction codes^15^ and the tradeoff between the writing and reading cost associated with DNA storage^16^. Nanopore sequencing has recently emerged as a promising technology for low-cost and real-time reading of encoded data^3,7,8^. However, it is subject to unique error profiles consisting of substantially higher rates of indels. Because of the higher error rates in nanopore sequencing, developing a robust data encoding scheme is a requirement when using this platform for DNA data reading.

**Figure 1:**
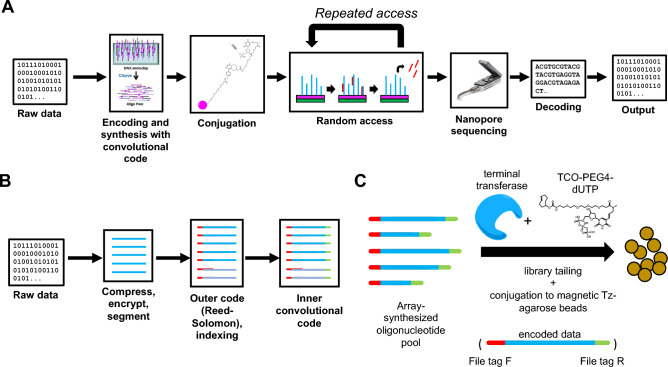
MDRAM overview. (**A**) Raw data is encoded into nucleotide bases using a convolutional encoding workflow, and are synthesized as an oligonucleotide pool. These fragments are then conjugated onto functionalized magnetic agarose beads, after which specific data elements can be repeatedly targeted by PCR. These fragments can be sequenced on the Oxford Nanopore system, after which they are decoded using an integrated workflow that does not rely on individual basecalled nucleotides. (**B**) Data encoding. Information is encoded with both an outer and inner code. The outer code (Reed-Solomon) accounts for oligonucleotide dropouts that may occur during synthesis. The inner code accounts for errors that may occur during the synthesis and sequencing processes. (**C**) Array-synthesized oligonucleotides are functionalized with *trans-*cyclooctene dUTP with terminal transferase, and then conjugated to methyltetrazine-functionalized magnetic agarose beads. Each oligonucleotide contains adapter sequences (red and green) such that subpools of oligonucleotides can be enriched by PCR.

Addressing the challenges of error correction and iterative random data access, we developed a highly scalable random access file system called magnetic DNA-based random access memory (MDRAM). This technology uses new advances in DNA encoding for maximum reading efficiency and repeated data readout operations. MDRAM has several fundamental innovations in the field of DNA data storage (Fig. 1A). This system involves covalently conjugating DNA data files, in the form of oligonucleotides, to solid-phase magnetic agarose beads. Some magnetic bead workflows operate in reverse: the target DNA is removed from solution by binding to magnetic beads while the overall pool remains in solution^17,18^. In our work, the original DNA data files are conjugated to the magnetic agarose beads and the targets are copied and eluted into solution. The result is that DNA data files can be selectively sequenced without losing the original molecular DNA media—this feature enables multiple repetitive read operations. We note that previously demonstrated methods for bead-based encapsulation of encoded DNA^19–21^ have in contrast been limited to single-use applications that prevent re-interrogation of source material. Other works utilizing magnetic beads to bind DNA-encoded oligonucleotides^22–24^ used non-covalent streptavidin–biotin coupling, which prevents the use of denaturing agents such as formamide to completely dissociate reaction products. On the computational front, we improve DNA encoding using convolutional codes that decrease the reading costs using noisy nanopore sequence reads by an order of magnitude—to levels comparable to high-accuracy Illumina sequencing reads. Uniquely, we directly work on the raw electrical traces rather than on basecalled reads to perform signal decoding and error correction. This feature leverages rapid nanopore sequencing readouts and thus enables low-cost, real-time data retrieval. Using MDRAM we also demonstrate a combinatorial barcoding system for labeling and retrieving data packets that can scale to exponentially large numbers of files. This enables a unique DNA targeting scheme that is exponentially scalable without the burden of individually synthesizing individual primer pairs for enrichment. Overall, we demonstrate significant improvements towards the implementation of robust encoding and scalable access of DNA elements. We demonstrate an end-to-end systems integration combining innovations in molecular storage, information encoding, and target enrichment.

## Results

### Encoding DNA with convolutional codes

In this study, we designed and optimized a schema for writing data into a DNA information payload with a convolutional encoding method optimized for nanopore sequencing^25^. Designed for use with nanopore sequencing, encoded oligonucleotides contain an inner code to mitigate sequencing errors and a Reed Solomon (RS) outer code^11^ to mitigate sequence dropouts (Fig. 1B). We employed convolutional coding as the inner code. A convolutional encoder encodes a stream of message bits into a sequence of encoded bits, which are computed as a linear combination of a past window of m input bits^25^. A convolutional encoder takes in a stream of input message bits and outputs the encoded bits. At any given time the encoder keeps a state consisting of the last m input bits. The encoded bits at a given time instant are computed as a linear combination (modulo 2) of the state bits and the current input bit, with the coefficients of the linear combinations chosen to maximize the error correction capability of the convolutional code. Each encoded bit can be considered as a checksum over the current window of input bits. The rate parameter r refers to the coding rate, which is the ratio of input to output encoded bits. As an example of how these two parameters influence DNA data encoding and decoding, let us consider an example where the convolutional code has the following value of m = 6 and r = 0.5; this produces 2 output bits per input bit. As m increases, the code becomes more powerful, but the decoding becomes slower due to an exponential increase in the number of possible decoded states. Moreover, each data sequence has a convolutional code and a cyclic redundancy check (CRC) error-detecting code^26^. Additional sequence elements incorporated in the synthesized oligonucleotides also consist of adapter sequences for targeted PCR amplification, which flank the internal index sequence and encoded data payload (Supplementary Fig. S1).

To test the chemistry underlying MDRAM media, we used an oligonucleotide pool denoted as Pool A. This set of contains ~ 12,000 unique array-synthesized oligonucleotides spanning 13 subpools (‘files’) that can be targeted by PCR. The sequences encoded a collection of various song lyrics, speeches, and the Universal Declaration of Human Rights totaling a size of 11.3 KB. Each of the 13 subpools encoded the same compressed archive of the aforementioned material using an early implementation of the convolutional encoding scheme, using a variety of m and r parameters; Pool A’s encoding performance was described previously^25^.

### Stable retention of DNA data elements onto magnetic agarose beads

Encoded DNA was functionalized by adding *trans*-cyclooctene (TCO) modified dUTPs with terminal transferase. This enzymatic step enables the conjugation onto customized methyltransferase (Tz) functionalized magnetic agarose beads—the chemistry involves an inverse electron demand Diels–Alder cycloaddition (Fig. 1C). Amongst all variations of click chemistry schemes, the kinetic rate of TCO-Tz conjugation is particularly suitable for modifying molecular analytes at submicromolar concentrations^27^.

**Figure 2:**
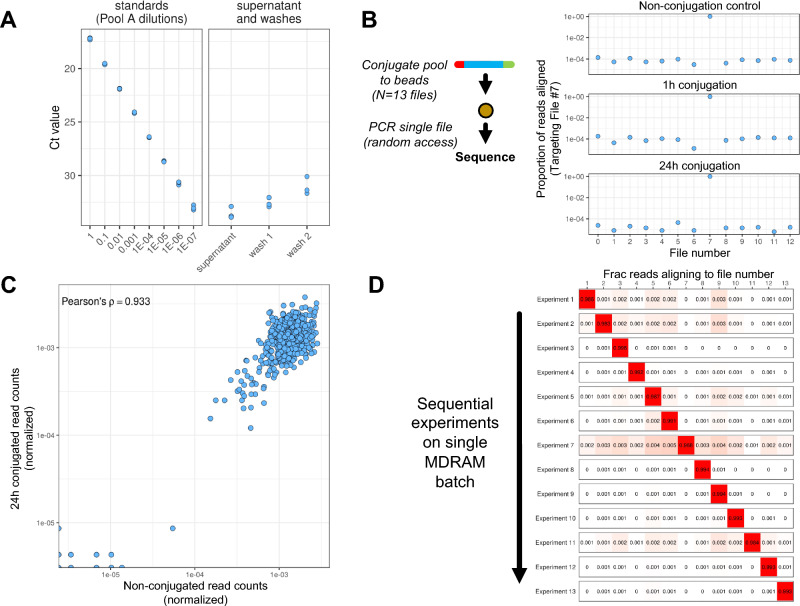
Random access of data elements with MDRAM. (**A**) Assessment of conjugation efficiency. qPCR was performed on the supernatant, which measures the amount of DNA not conjugated to the magnetic agarose beads and after wash steps. Left: Standard curve of DNA ranging several orders of magnitude. Right: quantitation of residual DNA from the conjugation reaction and through wash steps. Each dot represents a qPCR replicate. (**B**) Random access of a data element. A single DNA data file was amplified from MDRAM with different conjugation times (1 h and 24 h), and its on-target alignment rate was compared to an unconjugated control experiment with an aqueous DNA template. (**C**) Assessment of oligonucleotide abundances. The distribution of individual oligonucleotides from a targeted file was measured against the unconjugated control. The Pearson correlation coefficient was 0.933. (**D**) Iterative and sequential random access of data elements. Individual data files were sequentially retrieved from MDRAM. In between each experiment, the beads were washed, after which another file was targeted. The on-target alignment rate is shown for each random access experiment on the same bead batch.

First, we evaluated the efficiency of the conjugation chemistry with a variety of experiments. This step involved conjugation reactions with 70 nanograms of Pool A by adding TCO-functionalized dUTPs via terminal transferase^28^ and then mixing the product with Tz-functionalized magnetic agarose beads. After the conjugation reaction, we separated and collected the supernatant of the original reaction. We used fluorimetry to quantify the amount of remaining DNA left in the supernatant after the conjugation reaction. The amount of remaining DNA was below the limit of detection, indicating that the majority of DNA was conjugated onto the magnetic agarose beads. To verify this fluorimetry result, we performed qPCR on the supernatant of the conjugation reaction and the eluent from different washings of the MDRAM beads. As a measure of residual DNA material left in the supernatant and washes, we quantified the Ct value corresponding to a single file from the oligonucleotide pool and used a serial dilution of the oligonucleotide pool stock as the quantitative standard. Less than 0.01% DNA material was detected in the conjugation supernatant and in each wash step (Fig. 2A). These results indicated a near complete, stable conjugation of the DNA material to the magnetic agarose beads with minimal loss of material during wash steps. This high level of efficiency is consistent with other published measurements of Tz-TCO’s conjugation efficiency with biomolecules^27^.

### Retrieval of data elements from MDRAM

To demonstrate random data access capability, we performed targeted sequencing of specific data files from the MDRAM substrate containing Pool A. To assess the overall performance of DNA amplification from MDRAM we used sequencing to determine the on-target rates of a single DNA data file (e.g. number 7) (Fig. 2B). Two different bead conjugation conditions were tested; one involving a one hour incubation and the other for 24 h. We performed PCR reactions across two different bead conjugation conditions, sequenced the resultant amplicons on the Illumina platform and counted reads using the entire multi-file oligonucleotide pool as the alignment reference (Fig. 2B, Supplementary Table S1). Illumina-based sequences were used to accurately assess the count distribution of the amplified individual oligonucleotides. As a control, we compared the MDRAM read counts versus those amplified from a baseline in-solution oligonucleotide pool from the original array synthesis. Referred to as the non-conjugation control, this comparison sample involved direct sequencing from PCR amplification of the aqueous oligonucleotide pool. This control did not use the MDRAM media.

We aligned all sequenced reads using the designed oligonucleotide sequences as a reference. The proportion of on-target reads were over 95%. We noted a similar performance between two different conjugation times when compared to non-conjugated controls (Fig. 2B). This result confirmed that reading specific data files is done with high efficiency when the template DNA is conjugated to magnetic agarose beads. We measured the count distribution of detected oligonucleotides against a non-conjugated control and determined the concordance with MDRAM-amplified product. We observed a Pearson correlation coefficient of 0.933. This result indicates that read distributions were not significantly affected when conjugated to magnetic agarose beads versus the in-solution oligonucleotides (Fig. 2C).

### Sequential random access of DNA data elements with nanopore sequencing

MDRAM is a stable storage medium that enables sequential reading of different data files from the same substrate. Using PCR amplification, we performed serial random file access operations for each of the 13 files of Pool A on the same MDRAM media. For this test, we conjugated 70 nanograms of a DNA data oligonucleotide pool A to a new batch of magnetic agarose beads. An Oxford MinION instrument was used to sequence the amplicons. Between each experiment, the beads were washed before PCR amplification for the next file was performed (“Methods”).

The sequences were aligned to a reference representing the synthesized oligonucleotide sequences. The on-target rate of accessing each file was over 98% for all sequential read operations with each PCR primer pair exhibiting a minimum observed on-target rate of 96.8% (Fig. 2D, Supplementary Table S2). We measured the off-target sequences (i.e. reads aligning to file 1 when targeting for file 2). There was less than 0.1% of crossover contamination from the other DNA file products across all experiments. This result indicates that MDRAM can be used for sequential read operations of specific data files without contaminating reads from other files or previous read iterations.

### Accurate convolutional decoding integrated into nanopore basecalling

To improve the accuracy of reading MDRAM files with nanopore sequence, we developed a decoding workflow that can be integrated with open-source nanopore basecaller software (“Methods”)^25^. For this study, we used the Bonito basecaller^29^, which processes nanopore signal data using neural networks. In this approach, information is drawn directly from the nanopore raw electrical current signals rather than from basecalled reads—the latter contains artifactual substitutions, insertions, and deletions in the sequences, some of which arise because of nanopore signal fluctuations. Convolutional decoding^30^ enables serial error correction defined by a state transition diagram; the decoding is thus performed efficiently using a dynamic programming-based Viterbi algorithm. In the Bonito basecaller framework, upstream of the basecalls are probability scores that can be integrated with state transitions in the convolutional decoding framework. The output is then a list of top candidate codewords, denoted as L and a default value of 8. Additional filtering comes from the CRC technique^26^ which provides added error detection and allows one to discard reads under circumstances where the convolutional code makes an error. Full details and source code are described in “Methods”.

We measured the performance of our convolutional encoding and decoding scheme across multiple encoding parameters. We used an oligonucleotide pool (‘Pool B’) with approximately 15,000 non-overlapping sequences. Pool B had an expanded number of data files compared to Pool A, with a 12.7 KB dataset used for each parameter setting. In comparison with Pool A, Pool B focused on higher rate and lower memory codes to test the limits of convolutional coding, and to move towards practical systems with lower writing costs and computational requirements (Supplementary Table S3). Also, Pool B incorporated additional encoding strategies and was synthesized using high-fidelity array synthesis chemistry from Agilent^31,32^. Details of the additional files are listed in Methods. Each data file, corresponding to an oligonucleotide subpool, was PCR amplified with primers flanking the file of interest (Supplementary Table S4) and underwent nanopore sequencing (Supplementary Table S2).

We analyzed the decoding performance as influenced by two encoding parameters, the convolutional code memory (m) and rate (r) on a random subsampling of reads (N = 10 trials). We assessed the percent of successfully decoded reads for the different values of m and r, meaning whether the sequenced data successfully decoded back to the file of interest (Supplementary Table S5). As expected, we observed that a higher memory and lower rate consistently led to a greater fraction of successfully decoded reads (up to 88%) at the cost of more computation and higher writing cost (Supplementary Fig. S4). Interestingly, the results were not monotonic; there was a general trend with codes having a m = 11 and with r = 3/4 associated with a lower reading cost. The overall improvements were substantial, leading to more than two times higher decoding success rate in some cases (Supplementary Table S6) when compared to our prior results^25^. The improvement is more pronounced for higher rate and lower memory codes; these settings enable the use of more practical and efficient codes while achieving similar performance to more computationally intensive and lower density codes. Across the different subpools, we also explored other optimization strategies, such as utilizing multiple CRC segments and finetuning the Bonito basecaller model but did not observe significant nor consistent improvements in decoding performance (Supplementary Fig. S5, Supplementary Text).

**Figure 3:**
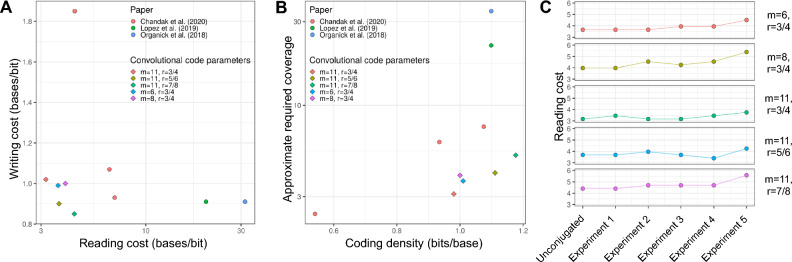
Efficient encoding of information in DNA with convolutional codes. (**A**) Convolutional code performance. The reading and writing cost of the convolutional code was measured for a variety of parameters and compared to other works. (**B**) Approximate required sequencing coverage. The amount of sequencing reads in order to successfully decode a file is shown as a function of the coding density (bits/base) and compared to other works. (**C**) Application of convolutional codes to parallel file access in MDRAM. Multiple files were accessed by MDRAM in parallel by splitting beads and performing PCR. Beads were then pooled together, washed, and split again for another experiment. The reading cost is plotted as a function of experiment number.

We measured other performance metrics that included: writing cost in terms of bases synthesized per information bit; reading cost measured in bases sequenced per information bit; and minimum required read coverage associated with each experimental parameter. Prior reports of DNA data storage^7,9^ described the coding density (inverse of writing cost) and coverage (reading cost divided by writing cost). We converted these metrics to the ones mentioned above for comparison (“Methods”). Previously, we described^16^ that reading cost is a better representation of the actual cost of sequencing compared to coverage, especially when comparing schemes with unequal writing cost or coding density. We considered the well-performing m = 11 codes across all rates (r) alongside selected rates for m = 6 and m = 8 to simplify the analysis (Fig. 3A). Consistent with the results measuring the proportion of correctly decoded reads, we observed significantly improved reading costs with our convolutional encoding/decoding scheme. Specifically, there were 2–3 × lower reading costs compared to our previous work with nanopore sequencing^25^. We achieved a reading cost of 3–5 bases/bit, compared to 22 and 34 bases/bit achieved by previously published strategies^7,12^ for nanopore sequencing based readout. These results indicated a cost that was 10 times lower than other encoding frameworks—we attribute this to reduced reliance on consensus for error correction that was used by the other methods (Fig. 3A).

For successful decoding in other frameworks, approximately 30-fold coverage is needed^7^. In contrast, our strategy required as low as threefold read coverage from nanopore-based sequencing (Fig. 3B). Most striking is that for writing costs of one base/bit, our nanopore-based framework not only improved on our previous work^16^, but also is competitive with other works^7,9^ that developed coding methods for Illumina sequencing (Supplementary Table S8). This improvement with nanopore sequencing was notable given its substantially lower basecalling accuracy (~ 5–10%) compared to Illumina sequencing (~ 0.1–1%). This result also points to the strength of convolutional coding and the basecaller-decoder integration framework.

### Repeatable, parallel, and multi-file access on magnetic agarose beads

We leveraged our improved encoding scheme for highly efficient and parallel random access operations on magnetic agarose beads. MDRAM enables multiple parallel random read operations by splitting the bead media. Afterwards, the bead substrate is washed and combined for subsequent data access operations. To demonstrate MDRAM’s performance over multiple iterations, we conducted random access reading on specific subpools. Here, we conjugated 100 nmol of Pool B onto magnetic agarose beads. We split the magnetic agarose beads into five wells and in each well we targeted a different file using a paired PCR primer pair specific to that data file (Supplementary Table S2). After amplification, the beads were pooled back together and washed for a new experiment.

We assessed the performance of integrating our improved convolutional coding onto MDRAM beads. This part of the study involved included a non-conjugated control—the original aqueous oligonucleotide pool—for every targeted file of interest in every flowcell to control for run-to-run variation in sequencing quality. Base quality variation was apparent among the different sequencing runs with the mean basecalling error rate varying from 4.5 to 7%. However, there were no significant differences in base quality between the MDRAM versus non-conjugated libraries when sequenced in the same flowcell (Supplementary Table S7A).

We also evaluated whether there were substantial decoding impacts on the MDRAM platform. The outer Reed Solomon code requires a specific minimum number of unique sequences to be correctly decoded. In cases with significant coverage variance or dropout of certain sequences, a higher number of reads is required for decoding the data. We determined that the read coverage variance was higher for the MDRAM-based experiments, leading to a higher reading cost required compared to the aqueous oligonucleotide pool template control (Supplementary Table S7B,C). However, the convolutional code parameters with more powerful error correction capabilities robustly controlled this effect. The code with highest memory (m = 11) and lowest rate (r = 3/4) had only a 15% variation in reading cost across repeated data readout operations (Supplementary Table S7D,E). The decoding performance across iterative read operations had less than 10% variation in the proportion of successfully decoded reads (Supplementary Table S7F). When using MDRAM, the reading cost across iterative random access operations remained up to an order of magnitude lower compared to recent nanopore-based DNA storage methods (Fig. 3C, Supplementary Table S8). The total sequencing yield also remained consistent between experiments, indicating that DNA was not substantially lost (Supplementary Table S2). Overall, when using MDRAM for repeated access operations, the reading cost of our new encoding framework remained below 5 bases/bit in all but two data points, and below 4 bases/bit for the m = 11 and r = 3/4 encoding parameters.

### Exponential-scale hierarchical file access

We developed a strategy for improved data access that is scalable to large numbers of data file elements with MDRAM. Read operations that access data elements using conventional PCR are severely bottlenecked by the design and synthesis of individual primer pairs to amplify and read a given file of interest. In a conventional random access scheme where individual data elements are accessed by a PCR amplification, the number of primers scales directly with the number of files. In conventional computer-based file systems, there may be millions of files that are all accessed individually. In a DNA-based storage system, the conventional PCR-based approach for selectively amplifying specific files is restricted in its scalability. It is nearly impossible to synthesize the number of PCR primers required to sequencing all the various files. Another challenge is that a significant number of primer pairs will have off-target amplification for any given file of interest.

**Figure 4:**
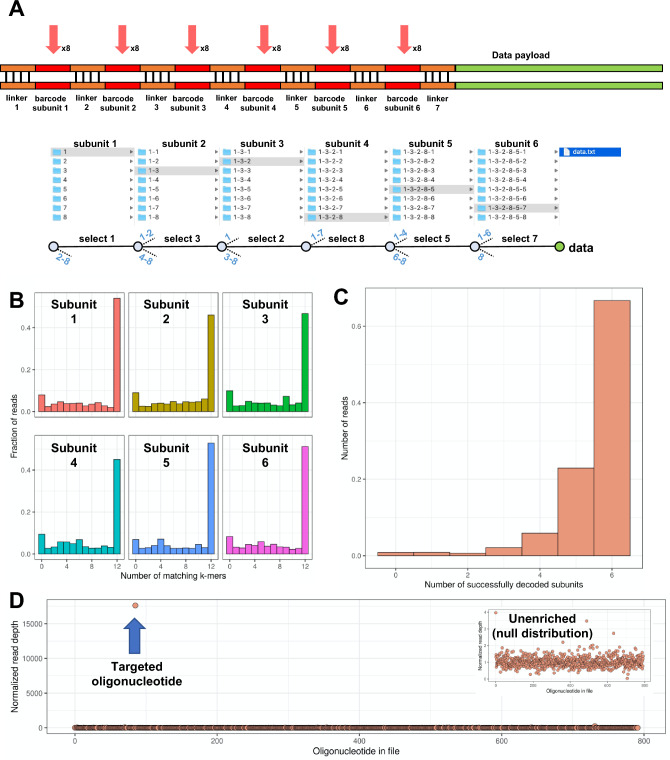
Massively parallel file barcoding with MDRAM. (**A**) Structure of combinatorial barcode addresses (CBA) for tagging data elements. A combinatorial barcode subunit and scaffold structure creates unique indexes for retrieving data elements. This creates a prototype hierarchical filesystem structure whereby individual data elements can be accessed by using primers sequentially targeting individual subunit locations. Bottom: a mock system relating CBA random access to filesystem tree traversal. (**B**) CBA sequence determination with nanopore sequencing. A k-mer matching scheme was used to determine the CBA sequence without alignment. The number of matching k-mers to the best match is shown for each subunit location. (**C**) The number of CBA sequences with perfect matches (no errors) is shown. (**D**) Targeting of a specific CBA to enrich for an oligonucleotide of interest. The median-normalized read count is plotted for each oligonucleotide for File 5 in Pool B. The arrow indicates the oligonucleotide of interest. Inset: The null distribution from an unenriched (non-targeted) pool of CBAs for File 5.

Providing a solution for this constraint, we improved the read operation process with a hierarchical file system for MDRAM. We refer to this scheme as combinatorial barcode addresses (CBAs). With it, an exponentially scalable set of different data elements can be tagged and accessed with only a small number of oligonucleotide sequences (Fig. 4A). CBAs use a highly diverse combinatorial schema for sequence tagging and retrieval; it has similarities to semiconductor-based demultiplexer schemes that connect binary addresses to devices or signals. As a proof of concept, we designed a scaffold structure consisting of six barcode subunits (‘bit positions’), each of which can have eight possible states as represented by a uniquely identifiable oligonucleotide sequence (octal, or base-8). In order to be robust against sequencing erorrs, these uniquely identifiable oligonucleotide sequences are pre-designed to have large edit distance between them. Random selection of one of these eight sequences in the six subunit positions resulted in 262,144 independent barcodes that are accessible with only 48 oligonucleotides (Supplementary Table S9). Each oligonucleotide sequence consists of a barcode (20 bp) flanked by two adapter sequences (20 bp each) that are required for combinatorial assembly into a larger barcode structure. For applicability to nanopore sequencing, we designed the barcode sequences to be uniquely identifiable using unique k-mer sequences (“Methods”).

CBAs enable scalable random access of individual data elements. Random access is performed using a series of amplification steps with a small number of different primers. This sequential process enables traversal through the file hierarchical tree and reading specific DNA elements of interest (Supplementary Fig. S6). Initially, the DNA template consists of a pool of CBA-data element structures. To access an arbitrary data element, six sequential amplification reactions targeting each barcode subunit are performed, where the product of one reaction is diluted then used as the template for the next. Each amplification reaction traverses one subunit level of the CBA structure. The product from one amplification reaction is purified, diluted, and used as template for the next reaction. After six targeting reactions, the final amplicon belonging to the CBA-data element payload of interest is read through sequencing. For example, a primer corresponding to a barcode on subunit 1 was used in an amplification reaction. This amplifies sequences containing that barcode at subunit 1, resulting in enrichment of the nested elements in the hierarchical tree. The amplification product was purified and used for the template for the second amplification reaction with another primer corresponding to subunit 2, and so forth. Eventually, amplification for a sequence at barcode subunit 6 results in the data element of interest. All primers are listed in Supplementary Table S9.

To implement and validate CBA chemistry, we tagged individual oligonucleotides belonging to File 5 from Pool B with random CBAs. We amplified File 5 from Pool B and used the amplified DNA for random assignment of CBA addresses across the represented amplicons. We performed a one-step Gibson Assembly^33^ reaction between amplicons of File 5 and a pool of all CBA scaffolding oligonucleotides (Supplementary Table S9) to generate a pool of CBAs, composed of randomly tagged oligonucleotides (“Methods”). The CBA structure and the data elements are joined together by a linker sequence (Fig. 4A, Supplementary Table S9). After the Gibson Assembly reaction, the products were amplified, cloned into topoisomerase-I activated vectors and transformed into *E. coli* to control for the overall library diversity.

To determine how individual oligonucleotides were associated with a given CBA, we nanopore sequenced the PCR products inserted into the vector. Lawns of bacterial colonies were pooled, amplified by PCR with a set of common primers, and sequenced to determine the oligonucleotide data payload for each CBA. This serves as a lookup table similar to a filesystem tree used in computing systems. To perform address decoding, we used a k-mer matching algorithm that searches for unique sequencing that belong to each possible barcode at each subunit position. For each read and subunit position, we measured the number of matching k-mers (k = 9) to every possible designed barcode sequence. Overall, we observed that ~ 30–40% of sequences in each subunit had a perfect match to the designed barcode sequence (Fig. 4B). As each barcode sequence consists of k-mers that are uniquely distinguishable from one another, sequences that had a less than perfect match can still be resolved. Overall, the entire CBA was successfully decoded for ~ 70% of sequenced reads containing a CBA tag (Fig. 4C) which translates to an unrecoverable failure rate of approximately 6% at each barcode subunit, assuming that the errors between barcode subunits are independent.

Having determined the CBA distribution, we incorporated the CBA-File 5 constructs in the MDRAM format. Access of individual data elements was then performed on MDRAM by targeting for a specific sequence on each sequential barcode subunit (Fig. 4A). The first amplification reaction was performed using primers corresponding to the common flanking sequences (Supplementary Tables S4, S9), after which subsequent CBA traversal reactions were performed in aqueous solution (Supplementary Fig. S6). From our prior sequencing analysis of the original CBA pool, we counted 92,388 unique CBAs that are randomly linked to oligonucleotides from File 5 in Pool B. This number was determined using an inclusion criteria where k-mer scores for all other candidate barcodes for a given subunit must be zero. Using these CBA identities, we traversed the hierarchical tree with recombinase-based isothermal amplification^34^ (“Methods”). The targeting process involved six sequential amplification reactions—each amplification step uses a separate primer corresponding to the barcode sequence 5-11-23-29-39-48 and the reverse primer for File 5 (Supplementary Table S9). After completion of these serial amplification steps, we sequenced the resulting amplicon. There were 17,600-fold more reads corresponding to the targeted oligonucleotide of interest compared the median coverage across all other File 5 oligonucleotides (Fig. 4D) and an approximately 10,900-fold enrichment versus the null unenriched distribution (Fig. 4D, inset). Through this efficient and accurate targeting process, our results show a promising proof-of-concept for exponentially scalable random access of data elements on the MDRAM platform.

## Discussion

In this work we developed MDRAM which is an end-to-end framework for storage of DNA data elements. This DNA data storage method features high-fidelity and repeated access of synthesized oligonucleotides, as well as efficient decoding of sequence features using a convolutional encoding/decoding scheme. This DNA data technology utilizes an efficient click chemistry scheme to attach synthesized DNA onto functionalized magnetic agarose beads. We demonstrated MDRAM’s capacity for conjugation of data-encoded oligonucleotides. Importantly, the chemistry itself is widely applicable for conjugation of other types of DNA onto the magnetic substrate—for example, we anticipate that longer strands of information-encoded DNA^6^ or those from enzymatic synthesis^35,36^ would also be compatible with conjugation. Therefore, MDRAM is amenable for any type of biomolecule storage platform that is compatible with TCO-Tz labeling schemes.

We demonstrated that pools of data encoded oligonucleotides and their associated files can be selectively amplified from MDRAM over may iterations with little carryover contamination. Sequential read operations consisting of targeted PCR amplification of different DNA data files are performed on the same batch of magnetic agarose beads with negligible amounts of sample loss. Aqueous solutions of DNA data files are consumed during any data readout operation and must be eventually exhausted or re-amplified. In contrast, MDRAM would potentially enable facile long-term repeated accessibility of data elements. While storing DNA in solution or in dried powder would afford a higher storage density, the fundamental consumption of DNA in the retrieval process results in eventual exhaustion of the original template. While PCR and other amplification methods can be used to regenerate the sample, skews and errors will be inevitably introduced; working with the original conjugated template molecules addresses all of these challenges. While a comprehensive study on the long-term stability of the MDRAM platform is beyond the scope of this proof-of-concept study, we demonstrated robust decoding and sequential access of individual file elements of up to tens of read operations over the span of weeks. The decoding process as detailed also enables quantifiable measurements of data integrity as the apparent reading cost would change; this would serve as a metric to use for future benchmarking studies. As storage mediums are typically characterized with a specific read/write cycle lifetime, we anticipate that this type of analysis would be valuable for assessing any type of DNA-based storage system.

We demonstrated the data readout capability of MDRAM using an improved convolutional encoding/decoding workflow. When compared to other established methods utilizing nanopore sequencing, we found an overall reading cost improvement of approximately an order of magnitude. However, we note that a direct head-to-head comparison across methodologies is difficult due to various confounding factors, ranging from the use of different oligonucleotide synthesis providers, different amounts of data encoded, different oligonucleotide lengths synthesized and the ongoing improvements in nanopore sequencing technology. However, generalizations can be made: some works utilized the now-discontinued 1D^2^ nanopore sequencing method to mitigate sequencing errors by reading the opposing strand^7,12^ but resulted in lower throughput compared to this work. Smaller file sizes were used in our study to focus on exploring multiple coding parameters and their impact on the overall reading cost. Smaller file sizes also effectively reduced the amount of indexing space within the synthesized oligonucleotides as compared to larger files. That being said, we anticipate has only a small effect on the overall reading and writing cost as the index size scales logarithmically with file size. Finally, we note that nanopore basecalling performance has improved dramatically, with current error rates of 4–5% compared to 10% as recently as 2019^37^, when other nanopore-based works were published^7,12^.

Our novel decoding workflow offers unique advantages over consensus-based schemes. By utilizing the rich soft information in the raw nanopore signal instead of working with basecalled reads, we sidestepped explicitly handling indel errors through consensus or an edit-distance code. Another encoding scheme called HEDGES explicitly attempts to comprehensively correct for multiple types of sequencing errors^38^, but has not yet been experimentally verified on nanopore sequencers. We note that some prior works directly utilized raw nanopore signals for classification of sequence reads rather than decoding^39,40^; however, to our knowledge this is the first work that leverages raw nanopore signals for complete decoding and error correction. The convolutional code is capable of correctly decoding a large fraction of reads without clustering or consensus, leading to an order-of-magnitude reduction in required coverage. Our resulting nanopore-based reading cost demonstrate a high level performance that matches state-of-the-art coding schemes based on high-accuracy Illumina sequencing platforms^16^. This equivalent performance is striking given that there is an order of magnitude difference in error rates between nanopore and Illumina sequencing. The framework has flexibility, seeing that it can be integrated into other neural network architectures as nanopore basecalling improves over time. Overall, the replication experiments utilizing MDRAM clearly demonstrate the robustness of the framework in effectively handling experimental variation due to coverage variance, dropouts, and the base quality of the sequencing run. Data could be encoded using a user’s choice of parameters optimizing on either decoding speed, writing cost, or robustness to errors.

Modern data storage systems utilize filesystems for random access of individual data elements amongst millions of files if not more. Individual primer synthesis and data access with conventional PCR does not scale to these numbers. In this work, we also demonstrated in MDRAM a proof-of-concept of an exponentially scalable data addressing technology called CBAs. By virtue of exponential scaling of combinatorial oligonucleotide assembly, we demonstrated individual readout of oligonucleotides amongst a pool of ~ 15,000 other sequences with combinations of targeting oligonucleotides that can fit onto less than one 96-well plate. CBAs scale exponentially, which enables orders of magnitude higher numbers of unique barcode tags as opposed to other combinatorial PCR methods^18,41^. CBAs can also be incorporated onto data payloads with pre-determined barcode identities as opposed to being randomly assembled in our study; this would obviate the requirement for bulk sequencing to generate the CBA lookup table but may necessitate the use of robotic liquid handling to operate at scale. This work demonstrates that it is possible to mimic a basic feature of conventional filesystems—scalable random read access—on a DNA-based platform. In fact, in combination with our reduced reliance on sequence consensus, MDRAM could potentially enable real-time decoding with nanopore sequencing. With careful choice of convolutional coding parameters (m = 6, r = 3/4 and L = 1), we achieved decoding times of 0.25 s per read while still achieving a reading cost of ~ 4.5 bases/bit for a writing cost of ~ 1 bases/bit—which is a 5 × lower reading cost than another reported consensus-based approach^7^. We also include the decoding speed for other parameters in Supplementary Table S10. We note that the outer Reed Solomon code operates in blocks and hence the block size should be kept small enough to reduce decoding latency but large enough to achieve reasonable erasure (sequence dropout) protection. Excluding the DNA synthesis process with an external vendor, the conjugation time of synthesized fragments can be performed in less than one day. CBAs could be assembled, conjugated, and sequenced in less than two days. Through a large-scale robotic MDRAM-to-sequencer integration and optimization of bioinformatic pipelines, we believe that the appropriate set of coding parameters can be used along with real-time basecalling to enable close to real-time repeated access and decoding of data as would be idealized in DNA-based data storage systems.

## Methods

### Synthetic DNA samples (data files), and primers

We encoded a compressed data file of size 12.7 kB. Pool A and B are oligonucleotide pools that encode tarred, compressed and encrypted text containing a variety of poems, speeches such as the Gettysburg Address, lyrics and a declaration about human rights. The files include:

- gettysburg.txt: The Gettysburg Address by Abraham Lincoln
- mlkjr.txt: “I have a Dream” by Martin Luther King, Jr.
- rickastley.txt: Lyrics of “Never Gonna Give You Up” by Rick Astley
- poems.txt: A collection of poems:
  - “The Road Not Taken” by Robert Frost
  - “Stopping by Woods on a Snowy Evening” by Robert Frost
  - “If” by Rudyard Kipling
  - “Fire and Ice” by Robert Frost
  - “The Tyger” by William Blake
  - “A Psalm of Life” by Henry Wadsworth Longfellow
  - “Sonnet 18: Shall I compare thee to a summer’s day?” by William Shakespeare
  - “I Wandered Lonely as a Cloud” by William Wordsworth
  - “To Autumn” by John Keats
  - “Abou Ben Adhem” by Leigh Hunt
  - “Gitanjali 35” by Rabindranath Tagore
  - “Sympathy” by Paul Laurence Dunbar
  - “Caged Bird” by Maya Angelou
  - “Nothing Gold Can Stay” by Robert Frost
  - “The Rime of the Ancient Mariner” (excerpt) by Samuel Taylor Coleridge
- unhr.txt: The Universal Declaration of Human Rights

Synthetic DNA was prepared with the parameters ‘m’ (Convolutional code memory), ‘r’ (Convolutional code rate) with Reed Solomon code redundancy (Supplementary Table S3). The specific procedure to encode data is described later in the “Methods” in “DNA encoding using convolutional codes”. DNA sequences were synthesized by CustomArray (Pool A) and Agilent (Pool B) as a pool of oligonucleotides (Supplementary Data S1 and S2). For read operations by PCR, primer sequences of length 25 were added on both sides of oligonucleotide sequences of interest to enable single file targeting. All sequences are also available at the URL https://github.com/shubhamchandak94/nanopore_dna_storage/tree/bonito/.

### DNA conjugation to magnetic agarose beads

Synthetic DNA was quantified by Qubit fluorescence (Thermo Fisher Scientific, Waltham, MA). 70 ng of Pool A and 100 nmol of Pool B was used for bead conjugation. The DNA pool was denatured in a total volume of 45 µl in 1× terminal transferase buffer (New England Biolabs, Ipswich, MA) with 1× (5 µl) CoCl_2_ additive (New England Biolabs, Ipswich, MA) for 5 min at 95 °C followed by ramping to at 4 °C at 100% ramping rate. The tailing reactions were performed in 50 µl volumes with 100 µM of TCO-PEG4-dUTP (Jena Bioscience, Jena, Germany) and 1 µl of terminal transferase enzyme (New England Biolabs, Ipswich, MA) for 1 h at 37 °C. After 1 h of incubation, the reaction was purified using 1.8× Ampure XP beads (Beckman, Coulter, Brea, CA) and was resuspended in elution buffer (10 mM Tris–HCl pH 8.0, 0.05% Tween 20).

Methyltetrazine-functionalized crosslinked agarose beads were custom ordered from Cube Biotech (Germany). 50 µl of tetrazine beads were pipetted into a new PCR strip tube and washed twice with 100 µl wash buffer (10 mM Tris–HCl pH 8.0, 0.05% Tween 20) with low retention pipette tips. Afterwards, purified TCO-tailed DNA library was added onto the magnetic agarose beads and incubated overnight at room temperature in a rotating mixer to prevent the beads from settling. After incubation, unconjugated library fragments were measured by Qubit. The conjugated DNA samples on magnetic agarose beads were washed with wash buffer. The conjugation supernatant and wash steps can be saved for qPCR quantification. The samples were maintained in a storage buffer (10 mM Tris–HCl pH 8.0, 0.1% Tween 20, 50% glycerol) at − 20 °C.

### Read access of files with PCR

Files were retrieved with PCR amplification using separate barcodes used as primers at 1 µM concentration in 1× KAPA HiFi HotStart ReadyMix (Roche). Random access was directly performed by PCR by combining the master mix solution with the DNA-conjugated MDRAM. Primer sequences to target Pool A were previously published^25^, and primers to target Pool B are listed in Supplementary Table S2. Amplification conditions were 45 s in 98 °C, 18 cycles of 98 °C for 15 s, 60 °C for 60 s, 72 °C for 60 s, and 72 °C for 60 s before a final hold at 4 °C. A magnetic bead separator was used to remove the amplified products from the beads. The supernatant containing amplified DNA libraries were purified using 1.8× volume of Ampure XP beads. The DNA-conjugated beads were washed twice by incubation of wash buffer 2 (98% formamide, 10 mM Tris–HCl pH 8.0, 0.5% SDS) at 65 °C with mixing, twice with wash buffer (10 mM Tris–HCl pH 8.0, 0.05% Tween 20), and stored with storage buffer for further use.

We also developed another cleanup step to eliminate carryover DNA between the reading of different data files. In later split-pool experiments involving repeated access of the same file, we further performed a high-stringency wash step consisting of digestion of pooled MDRAM beads with 1 µl each of Exonuclease I and Exonuclease III (New England Biolabs, Ipswich, MA) in NEBuffer 2.1 for 30 min at room temperature, followed by heat inactivation for 20 min at 65 °C. Exonuclease I and exonuclease III are nucleases that digest double-stranded and single-stranded DNA from the 3′ end. Because the MDRAM system conjugates DNA to the magnetic agarose beads at the 3′ end through the addition of TCO-functionalized nucleotides, the template DNA is protected from digestion. Consequently, carryover DNA fragments from the previous amplification is digested while the conjugated DNA remains protected. When accessing the same file repeatedly, we observed that this enzymatic digestion step eliminated the buildup of amplicons over successive read iterations. These beads were then washed twice with wash buffer (10 mM Tris–HCl pH 8.0, 0.05% Tween 20), after which the beads were stored in storage buffer at − 20 °C.

To sequence the amplified DNA, we performed end-repair, a-tailing, and ligation of sequencing adapters to the samples. For Illumina sequencing, DNA samples were subjected to end-repair and A-tailing process for 30 min at 20 °C and then 65 °C for 30 min with the Kapa HyperPrep workflow (Roche) in a thermal cycler, after which ligation was performed for 10 min at room temperature using Illumina UD barcoding adapters when sequencing using the Illumina platform. The ligation reaction was purified using 0.8× volume of Ampure XP beads under standard protocols. In the next step the library was amplified in 50 µl KAPA HiFi HotStart Readymix (Roche) with an Illumina primer mix containing P5 and P7 sequences. Amplification conditions were 45 s at 98 °C, 12 cycles of 98 °C for 15 s, 60 °C for 30 s, 72 °C for 30 s, and 72 °C for 1 min before a final hold at 4 °C. Amplified libraries were purified using 1.8× volume of Ampure XP beads. All amplified and purified libraries were quantified with Qubit analyzer and was sequenced on an Illumina iSeq with 50 pM loading concentration.

For sequencing with the Oxford Nanopore platform, end-repaired and a-tailed DNA from the Kapa HyperPrep workflow was directly ligated with the AMX nanopore sequencing adapters using the LSK109 sequencing kit (Oxford Nanopore Technologies). We used the Native Barcoding Kit from Oxford Nanopore Technologies for multiplexed experiments. The ligation reaction was purified using 0.8× Ampure XP beads under standard protocols, with the exception of washing the beads with SFB buffer (Oxford Nanopore Technologies) instead of 80% ethanol, and eluted in EB buffer (Oxford Nanopore Technologies). All of the library was then immediately sequenced with a MinION R9.4.1 flowcell on the MinION nanopore sequencer for 48 h.

### Sequence data processing and alignment

Illumina sequence reads were demultiplexed using bclfastq v2.20 (Illumina). Reads were aligned using bwa-mem v2.17^42^, using the designed oligonucleotide pools as a reference. Each entry of the reference corresponds to a single oligonucleotide that was synthesized, making up a total of over 10,000 entries per reference. To assess targeting efficiency, read counts aligning to each sequence was tabulated using samtools v1.10^43^.

Nanopore sequence reads were basecalled and demultiplexed using Guppy (Oxford Nanopore Technologies). Sequence alignment was performed using minimap2 v2.17^44^. The designed oligonucleotide sequences were used as the reference, making up a total of over 10,000 entries. To assess targeting efficiency, read counts aligning to each sequence was tabulated using samtools v1.10^43^.

### DNA encoding using convolutional codes

For nanopore sequencing, we use the coding scheme from Chandak et al.^25^ with significant improvements through integrated basecalling and decoding stages (Supplementary Fig. S1). A convolutional code is scheme whereby the input stream of message bits is transformed into a sequence of encoded bits in which the computing is performed over a linear combination of a window of size ‘m’ (memory), denoting a number of previously observed bits in the data stream. Codes with higher memory are more powerful at the cost of decoding complexity. As with standard error correcting schemes, the length of encoded bits is governed by the coding rate ‘r’. For example, for coding rates of 0.5, 2 output encoded bits are created for each input bit.

We extended the pipeline to work with recent nanopore basecallers such as guppy and bonito. In addition, we modified the read trimming mechanism for removing the adapter and barcode sequences. These improvements in the inner code decoding pipeline lead to substantial improvements in the accuracy, in some cases doubling the number of the reads that are decoded correctly. Another modification was related to the fact that the previous scheme discarded reads where the CRC check failed, hence wasting non-trivial fractions of reads for which the decoding produced messages were mostly correct but had a few localized errors. The presence of such cases can be ascribed to the bursty nature of the decoding errors for convolutional codes. Therefore, we modified the strategy to use two CRCs for each oligo, protecting the first and second halves of the read, respectively (Supplementary Fig. S3). During decoding, we used a message from the list as long as at least one half has been decoded correctly, leading to further reduction in the number of reads needed for successful decoding. However, we discovered that the two CRC strategy only led to marginal gains in practice (see Supplementary Text), and hence focused on the simpler one CRC strategy for this analysis.

Input files were compressed and encrypted before encoding so that the input to the encoder appears random and does not produce excessive homopolymers (e.g., GGGG) which causes previously observed synthesis and sequencing errors^25^. Each input file is split into individual segments that are encoded into identifiable oligonucleotides. During the encoding process, an index and an 8-bit error correcting CRC are appended to each segment. Afterward, the convolutional code with specific encoding parameters ‘m’ and ‘r’ are applied to the segments, and is then encoded into nucleotide space with a standard 2 bits/base encoding (eg. 00:A; 01:C, 10:G, 11:T). The encoded DNA sequences (including the sequencing primers) were of length around < 200 nt and were synthesized by CustomArray (Pool A) or Agilent under their ‘HiFi’ synthesis method (Pool B).

### Integrating viterbi decoding with bonito basecaller probabilities

We integrated data decoding into v0.1 of the Bonito basecaller^29^. Bonito is based on connectionist temporal classification (CTC)^45^, which is a technique commonly used in speech recognition to bypass the need for labeling each timestep in the audio to a phoneme/letter. Bonito utilizes a convolutional neural network that downsamples the raw signal and transforms it into a sequence of state probabilities at each time step, where the states are A, C, G, T and “blank” (“b”). Blank is a special state that allows the length of the basecall to be lower than the length of the probability vector. This state probability vector was decoded into a basecall in two ways:

1. Greedy: Here the most probable state is chosen at each time step and then a collapse operation is performed wherein repeated states are collapsed and blanks are removed. E.g., AAAbCCbCCCGGbbbTTT would be collapsed to ACCGT.
2. Beam search: This method exploits the fact that a single basecalled sequence corresponds to multiple state sequences due to the collapsing operation and the actual probability is the sum of probabilities all such state sequences. A set (beam) of candidate basecall sequences is maintained at each step, and each of them is extended at the next step. The size of the beam is kept constant, eliminating the lowest scoring sequences at each step.

For decoding the convolutional codes, we follow the beam search strategy, which is compatible with the list decoding used in Chandak et al.^25^ (Supplementary Fig. S2). For the Viterbi decoding, we have states corresponding to (convolutional code state, position in base sequence). At each time step, each state stores a list of L candidate message prefixes (where L = list size or beam size). Along with the message prefixes, its current score is also stored (more precisely, we store the score for the top L paths ending with blank and the top L paths ending with non-blank CTC state). At the next step, we add one character (CTC state), add (using logsumexp operation) the scores for all the ways new bits can be added to extend the message prefix and take the top ones to maintain the list of size L. At the end of the Viterbi decoding process, we report the L messages corresponding to the state (convolutional code state = end state, position in message = total length). Other details including puncturing for high rate codes are described in Chandak et al.^25^. We found that a list size of 8 provides close to optimal reading cost and higher list sizes do not provide much benefit. The increase in reading cost for list sizes below 8 is relatively small and the list size should be chosen based on the tradeoff between reading cost and computational complexity.

### Barcode removal during decoding

The barcode/primer sequence needs to be removed before the convolutional code decoding which only expects the convolutional codeword as the input. This was done by first performing the basecalling, identifying the ends of the barcodes and then using only the probability vector corresponding to the convolutional codeword for the decoding. While the decoder is resilient to small imprecisions (few bases) in the barcode removal, larger errors can lead to suboptimal performance. We improved the performance by introducing a trimming penalty parameter to ensure that trimmed barcodes are correctly detected, while still giving preference to complete barcode sequences in the reads. In addition, we also made some other adjustments including better handling of cases involving chimeric reads where the start barcode might be found relatively later in the read than is usually expected.

### Computation of writing/reading cost

The writing cost for each experiment was computed as $(file\,size\,in\,bytes\,\times 8)/(\#oligos\,total\,\times\,oligo\,length)$ while the reading cost was computed as $(file\,size\,in\,bytes\,\times\,8)/(\#reads\,for\,decoding\,\times\,oligo\,length)$. In both cases, the oligo length did not include the primer sequence length. For the works that report coding density or bits per base excluding primers, the writing cost was computed as the reciprocal of these quantities. For works that report minimum coverage required for successful decoding, we computed the reading cost as $coverage\times\,writing\,cost$.

### Design and generation of exponential-scale combinatorial-barcode addresses (CBAs)

We generated exponentially large combinations of unique barcode sequences using an established isothermal gene assembly method called Gibson Assembly^33^. Each barcode subunit is flanked by common sequences that is required for the joining of oligonucleotides via this enzymatic assembly method. To generate these barcode sequences we randomly generated a set of 20-mer sequences that did not share any 9-mer sequences with each other. Forty eight of these sequences were chosen as the barcode sequences and seven chosen to be the flanking scaffold sequences. Oligonucleotides had the structure of, for example, [scaffold1]–[barcode1]–[scaffold2]. These oligonucleotides were synthesized by IDT without HPLC or PAGE purification and pooled together. The sequences are listed in Supplementary Table S9.

To link data elements to CBAs, we amplified files from Pool B where one of the primers contained a 5′ CBA linking sequence (ATACGTTAGTTCGGCAGTAT) corresponding to the last scaffold sequence of the CBA and a forward primer of interest (e.g. File 5). Amplification was performed in 1× NEBNext Ultra II Q5 Master Mix (New England Biolabs, Ipswich, MA), 1 µM forward and reverse primer for the file, and 100 nmol of Pool B. Ramping conditions were 45 s at 98 °C, 18 cycles of 98 °C for 15 s, 60 °C for 60 s, 72 °C for 60 s, and 72 °C for 60 s before a final hold at 4 °C. Amplicons were purified with 1.8× Ampure XP beads with standard protocols. We then performed Gibson Assembly using 1X NEBuilder HiFi DNA Assembly Master Mix (New England Biolabs, Ipswich, MA), 1 pmol of amplicon product, and 10 pmol of the CBA oligonucleotide pool. The reaction was incubated at 50 °C overnight, after which it was purified with 1.8× Ampure XP beads. To enrich for full-length products, we performed a second round of PCR in 1× NEBNext Ultra II Q5 Master Mix using 1 µM flanking scaffolding primers (AGTGGAGTTCTCCGCATCAA and a reverse primer from a file in Pool B) with ramping conditions of 45 s in 98 °C, 18 cycles of 98 °C for 15 s, 60 °C for 60 s, 72 °C for 60 s, and 72 °C for 60 s before a final hold at 4 °C. These constructs were then purified with 1× Ampure XP beads under standard protocols. These amplicons were full length CBA-data structures whereby the data payload is an oligonucleotide sequence drawn from the array-synthesized oligonucleotide pool.

To control for the total library diversity, we performed TOPO cloning of the CBA-data constructs using the Zero Blunt TOPO PCR cloning kit (Thermo Fisher Scientific). TOPO-ligated vectors were then purified with 1.8× Ampure XP beads, eluted with water, electroporated into ElectroMAX DH10B *E. coli* cells (Thermo Fisher Scientific) and plated as a lawn onto Kanamycin-50 LB agar plates overnight at 37 °C. The next day, the lawn of cells were washed off with 2 ml LB broth. 10 µl of cells were diluted into 100 µl elution buffer (10 mM Tris–HCl pH 8.0, 0.05% Tween 20) and then lysed by incubating at 95 °C for 5 min. One µl of crude lysate was then amplified with 1× NEBNext Ultra II Q5 Master Mix using 1 µM flanking scaffolding primers with ramping conditions of 45 s in 98 °C, 18 cycles of 98 °C for 15 s, 60 °C for 60 s, 72 °C for 60 s and 72 °C for 60 s before a final hold at 4 °C. MDRAM conjugation to magnetic agarose beads is performed as above using 70 ng of DNA.

### Random access of CBAs

We first performed PCR on the MDRAM beads using 1uM primers consisting of a common flanking primer (AGTGGAGTTCTCCGCATCAA) corresponding to the first scaffold sequence and a corresponding reverse primer to File 5 in Supplementary Table S4. The beads were amplified with 1× NEBNext Ultra II Q5 Master Mix with ramping conditions of 45 s in 98 °C, 18 cycles of 98 °C for 15 s, 60 °C for 60 s, 72 °C for 60 s, and 72 °C for 60 s before a final hold at 4 °C. The supernatant was removed from the beads and each well was purified with 1.8× Ampure XP beads under standard conditions. The MDRAM beads were then washed with Exonuclease I and Exonuclease III as described above. The ground truth CBA and data payload information was generated by sequencing each amplicon on a R9 flowcell on the PromethION nanopore sequencer for 72 h. Reads were basecalled into FASTQ files using Guppy, and reads were split into CBA and data payload sections using cutadapt (v2.9). CBA sequences were determined using a k-mer scanning algorithm that counts for unique k-mers found in each designed CBA sequence. They are matched with their corresponding data payload (oligonucleotide sequence) using the alignment program bwa. The inclusion criteria for CBAs in the ground truth lookup table was that for all six subunits there were no other k-mers associated with any other candidate barcodes.

We traversed the hierarchical tree of the CBA structure using a series of isothermal amplification steps with different primers. From the ground truth data, we chose a specific barcode sequence to target that corresponded to barcode sequence 5-11-23-29-39-48 tagged onto File 5. From the amplicons directly generated from the MDRAM beads, we performed multiple rounds of barcoded amplification using the barcode sequence (Supplementary Table S4) and the reverse primer targeting the entire file. Amplifications were in 1× TwistAmp Basic reaction mix (TwistDX, United Kingdom) with 1 µM each primer, at 37 °C for 20 min followed by hold at 4 °C. The reaction mix was diluted five-fold before cleaning with 1.8× Ampure XP beads. The amplicon was then diluted 1:200 into the next amplification step traversing the hierarchical file tree. Sequencing libraries for the Oxford Nanopore platform were then generated using LSK110 chemistry. The amplicons were then sequenced using a R9 PromethION flowcell for 72 h with data analysis as above.

## Supplementary Information

Supplementary Information 1.

Supplementary Information 2.

Supplementary Information 3.

## Data Availability

Source code for generating oligonucleotide sequences and analyzing sequencing reads derived from our convolutional coding scheme can be found here: https://github.com/shubhamchandak94/nanopore_dna_storage/tree/bonito. Sequence reads are deposited to NCBI’s Sequence Read Archive at PRJNA758230.
