# Supplementary material for: Magnetic DNA random access memory with nanopore readouts and exponentially-scaled combinatorial addressing
Source: Sci Rep. 2023 May 25;13:8514. doi: 10.1038/s41598-023-29575-z (PMC10213054; doi:10.1038/s41598-023-29575-z)
Supplement: Supplementary file 1 — Supplementary Information 1. [file 41598_2023_29575_MOESM1_ESM.pdf]

Supplementary Materials for

Magnetic DNA random access memory with nanopore readout and exponentially-scaled combinatorial addressing

Billy Lau, Shubham Chandak, Sharmili Roy, Kedar Tatwawadi, Mary Wootters, Tsachy Weissman, Hanlee P. Ji

Correspondence to: genomics_ji@stanford.edu, tsachy@stanford.edu

This PDF file includes:

Supplementary Text

Supplementary Figs. S1 to S6

Supplementary Tables S1 to S10

Other Supplementary Materials for this manuscript include the following:

Supplementary Data S1. List of oligonucleotides for Pool A.

Supplementary Data S2. List of oligonucleotides for Pool B.

Supplementary Text. Additional convolutional code optimization strategies

We explored additional optimization strategies to improve the performance of our convolutional coding scheme.

Impact of additional CRCs

We examined the impact of the new two-CRC strategy on the reading cost. Recall that the two-CRC strategy aimed at increasing the utilization of the convolutional decoded output by using parts of the read when the complete read is not decoded correctly. In **Supplementary Figure 5**, we observed that the results were mixed and there were no benefits of this strategy in improving data read quality. The reasons for this might include the fact that the overhead in reading cost for sequencing the second CRC offset any benefits in terms of fewer encoded reads per encoded sequence. Overall, this result pointed to the two-CRC strategy did not have an advantage over the simpler one-CRC strategy.

Bonito model finetuning

We performed finetuning (training) of the Bonito basecaller neural network parameters to exploit the fact that the DNA storage datasets differ significantly from the biological DNA datasets in terms of the strand length and base composition, among other factors. We generally followed the guidelines provided by the bonito tool usage guide, with some modifications. We first split the pool according to the convolutional code memory and rate (based on alignment) to obtain the training/validation split. To prepare the training dataset, the raw current reading sequences were first basecalled and aligned to the ground truth oligo sequences. The resulting pairs of raw current sequence and the oligo reference sequence formed the training data which is used

to further train the pretrained model obtained from the Bonito basecaller (at a low learning rate to avoid overfitting). Some changes were made to the training pipeline:

- Reducing the chunk sizes during the training to be consistent with the oligo sequence lengths. Bonito typically uses chunk sizes of ~2000-4000 bases which is not suitable for this data since the oligo sequences are of length ~200.
- Modifying the evaluation metric to use global edit distance instead of local edit distance. Local edit distance metric computes edit distance between the read and the part of the oligo that the read aligns to, while the global edit distance is the edit distance between the read and the entire oligo. For our case, global edit distance is more suitable because we need accurate barcode detection for decoding, and training based on local edit distance leads to highly trimmed reads.
- Remove quality value and homopolymeric region filters while preparing the training data.

While we saw improved decoding accuracy on validation datasets within the same synthesis and sequencing pool, this did not generalize well and provided negligible improvements for the newly synthesized pool. Since the training did not generalize well to reads in different synthesis pools, we used the default bonito model for the final set of experiments.

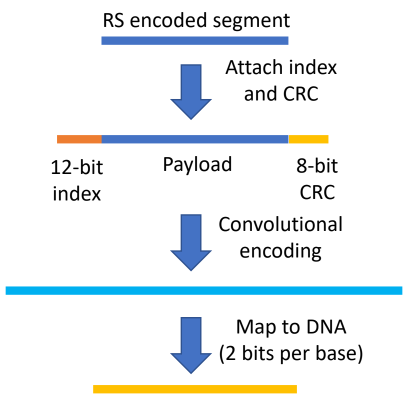

Supplementary Figure S1. Convolutional inner code encoding. Indexes and CRC are first attached to the RS segment from the encoder. Next, convolutional encoding is performed and is finally converted to a DNA sequence by performing the 2 bits per base mapping. Figure reproduced from (17).

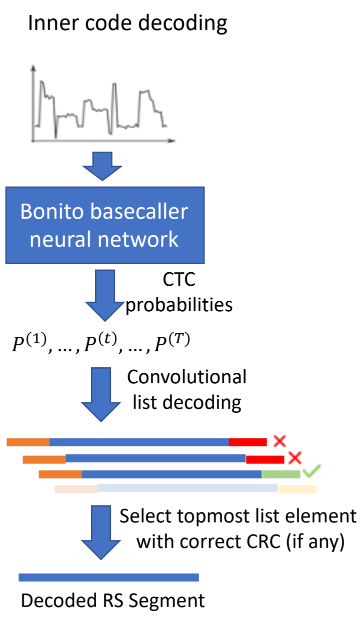

Supplementary Figure S2. Convolutional inner code decoding. The raw nanopore sequencing signal is passed through the bonito neural network to obtain the CTC probabilities, which are decoded using the Viterbi decoder which outputs a list of possible codewords. We select the highest scoring codeword that satisfies the CTC check, to obtain the decoded RS segment (to be used for outer code decoding later).

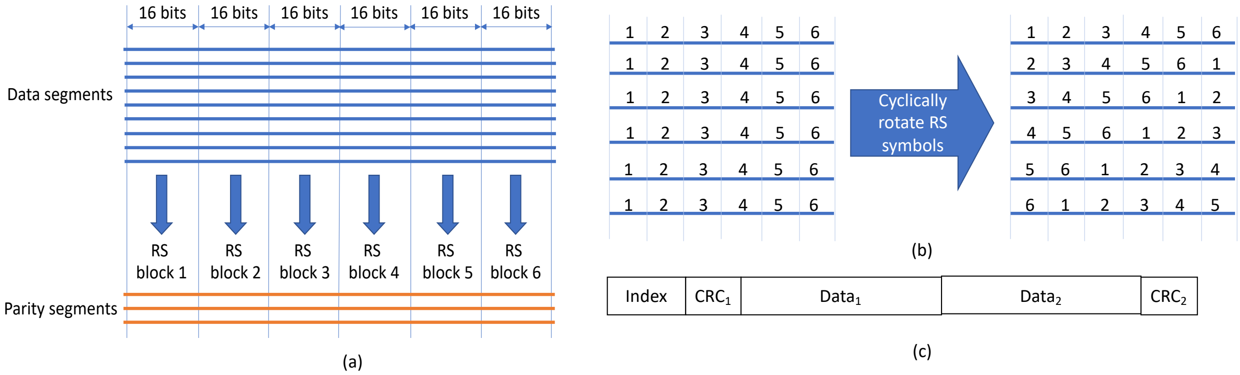

Supplementary Figure S3. Outer coding strategy. Two-CRC strategy for improving utilization of reads where only part of the read is correctly decoded. (a) RS encoding of the data based on Organick et al. (2018) and Chandak et al. (2020) (7,17). In this example, each segment is split into six 16-bit RS symbols and the RS encoding is performed in a columnar manner to generate parity segments. The decoding succeeds as long as the number of erasures and errors in each column after inner code decoding is sufficiently small. (b) The RS symbols in each segment are cyclically rotated in a predefined order for reasons explained later. (c) The input to the convolutional code. The first CRC protects index+data$_1$, while the second CRC protects index+data$_2$. Based on the observation that in sequences with correct index, data$_1$ is more likely to be correct (due to the bursty error characteristics), we perform cyclic rotation of the RS symbols in the segments to make sure that each RS block has roughly balanced number of correct symbols for decoding. Note that the decoding succeeds once we obtain sufficiently many correctly decoded symbols from distinct oligos for each RS code (marked 1 through 6 in figure (b)). Using two CRC sequences enables partial utilization of the reads where part of the message is successfully decoded.

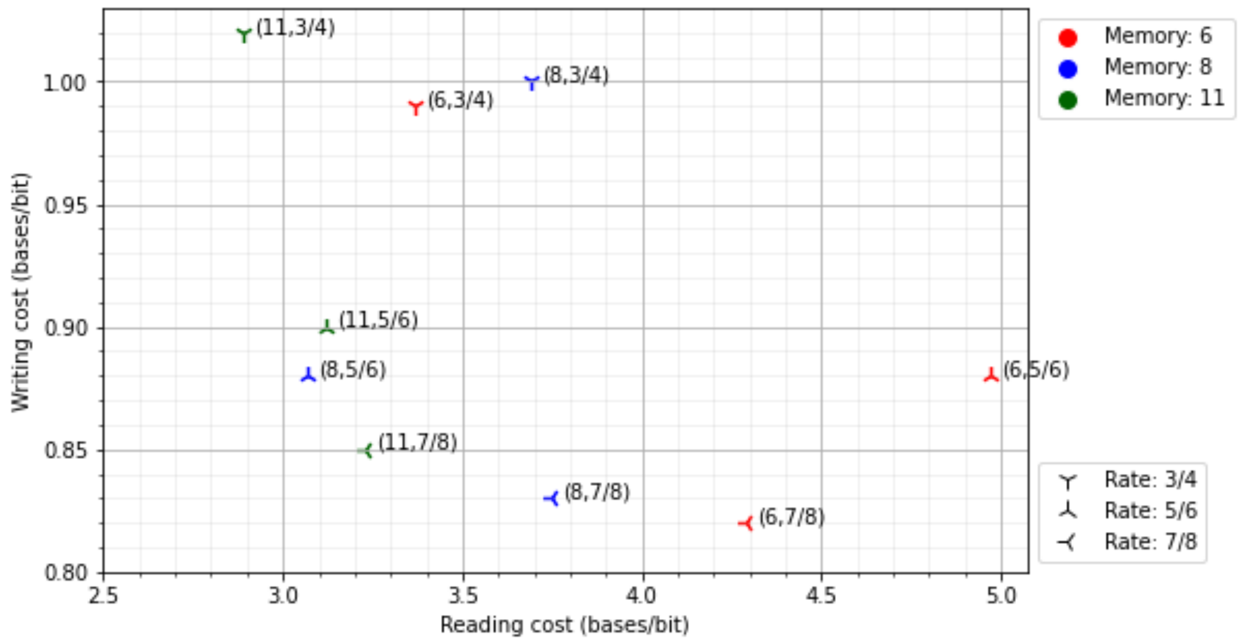

Supplementary Figure S4: Writing vs. reading cost across values of convolutional code memory (m) and rate (r). These results are for the subpools with the 1 CRC strategy. The small variation of writing cost with m (for fixed r) is due to the variation in the length of convolutional code padding bits.

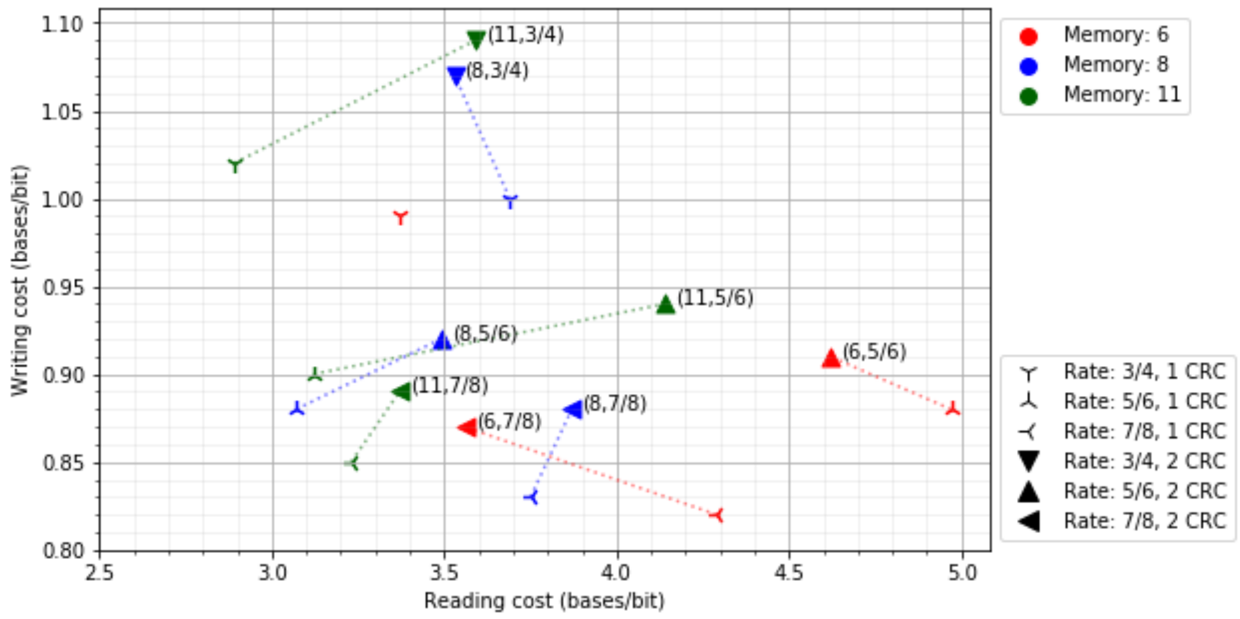

Supplementary Figure S5. Writing vs. reading cost across values of convolutional code memory (m) and rate (r), for the one-CRC and two-CRC strategies. The dotted lines connect the corresponding points for each (m,r) pair for clarity. The m=6, r=3/4, two-CRC subpool is not represented because of insufficient reads obtained in the amplification and sequencing process.

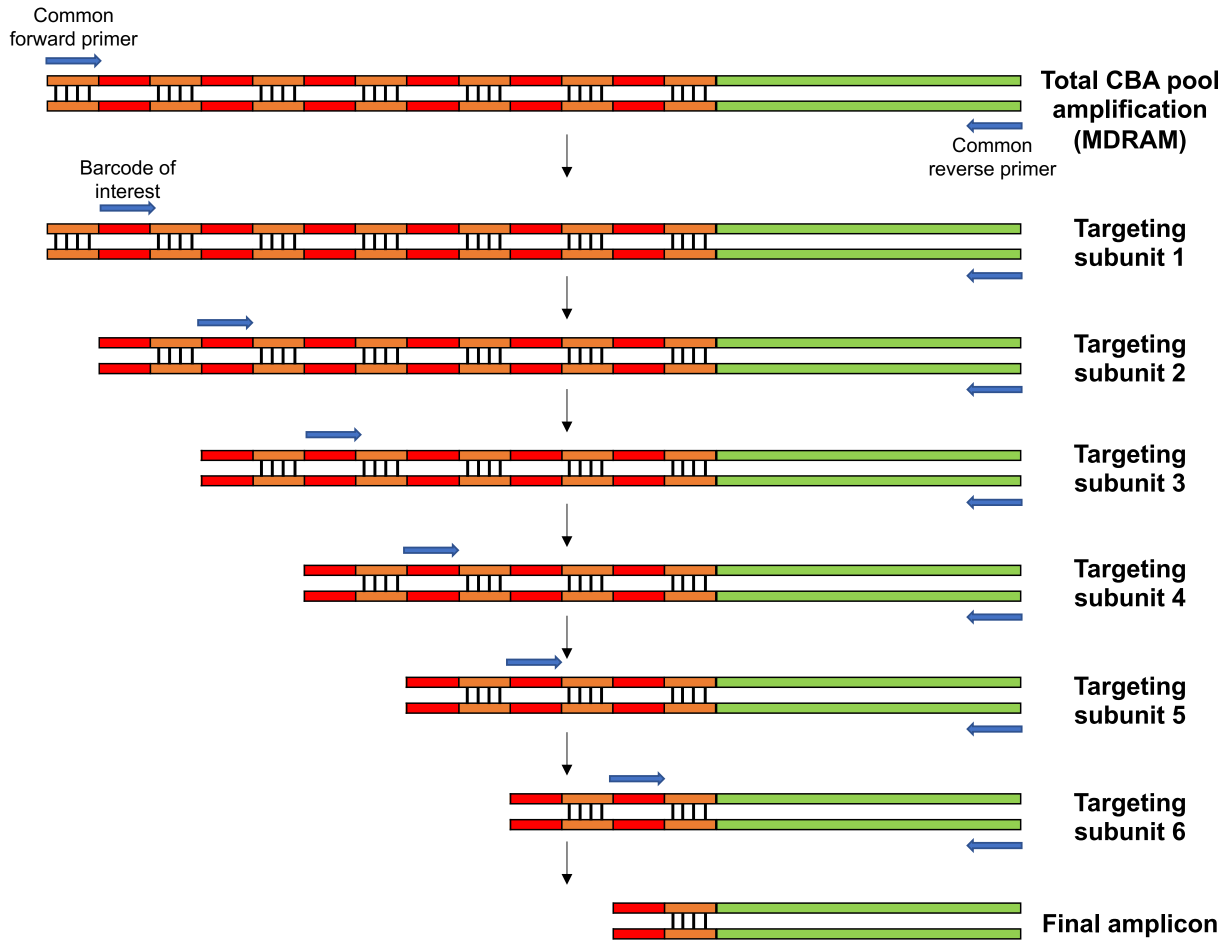

Supplementary Figure S6. CBA traversal with sequential amplification reactions. Primers corresponding to barcodes of interest are sequentially used in amplification reactions to traverse the CBA structure. Initially, the DNA template consists of a pool of CBA-data element structures. The product from one amplification reaction is purified, diluted, and used as template for the next reaction. Each amplification reaction traverses one subunit level of the CBA structure. After six targeting reactions, the final amplicon belonging to the CBA-data element payload of interest is retrieved. When used in the MDRAM format, total CBA pool amplification can be first performed by amplification using common flanking primers.

Supplementary Table S1. Sequencing Metrics (Illumina)

Oligo Pool	File	Experiment	MDRAM-conjugated	Iteration	# Reads	% aligned
Pool A	8	Fig 2B,C	No	1	323k	90%
Pool A	8	Fig 2B - 1h	Yes	1	562k	89%
Pool A	8	Fig 2B,C - 24h	Yes	1	348k	66%

Supplementary Table S2: Sequencing Metrics (Nanopore)

Oligo Pool	File	MDRAM-Conjugated	Experiment	Iteration	# Reads	% aligned
Pool A	1	Yes	Fig 2D	1	207.8k	89%
Pool A	2	Yes	Fig 2D	2	257.6k	89%
Pool A	3	Yes	Fig 2D	3	1044.9k	89%
Pool A	4	Yes	Fig 2D	4	451.0k	88%
Pool A	5	Yes	Fig 2D	5	257.3k	90%
Pool A	6	Yes	Fig 2D	6	283.9k	91%
Pool A	7	Yes	Fig 2D	7	78.9k	80%
Pool A	8	Yes	Fig 2D	8	665.0k	81%
Pool A	9	Yes	Fig 2D	9	625.6k	89%
Pool A	10	Yes	Fig 2D	10	481.0k	89%
Pool A	11	Yes	Fig 2D	11	152.0k	88%
Pool A	12	Yes	Fig 2D	12	504.2k	89%
Pool A	13	Yes	Fig 2D	13	832.9k	87%
Pool B	0,1,2,5,8	No	Fig 3A,B	1	9994.8k	94%
Pool B	0,1,2,5,8	Yes	Fig 3C	1	813.6k	93%
Pool B	0,1,2,5,8	Yes	Fig 3C	2	844.0k	94%
Pool B	0,1,2,5,8	Yes	Fig 3C	3	588.1k	93%
Pool B	0,1,2,5,8	Yes	Fig 3C	4	1813.0k	93%
Pool B	0,1,2,5,8	Yes	Fig 3C	5	1071.6k	87%
Pool B	0,1,2,5,8	No	Fig 3C	1	895.0k	90%
Pool B	5	Yes - CBA	Fig 4B,C	1	2449.2k	91%

Supplementary Table S3: Encoding parameters for each synthetic DNA subpool in Pool B

Subpool #	m	r	Synthesis vendor	filesize (bytes)	# oligonucleotides (data)	# oligonucleotides (Reed Solomon)	# oligonucleotides (total)	bytes per oligo	CRC length	message length	oligonucleotide length
0	6	0.75	Agilent	12672	704	176	880	18	8	165	114
1	8	0.75	Agilent	12672	704	176	880	18	8	164	115
2	11	0.75	Agilent	12672	704	176	880	18	8	164	117
3	6	0.83	Agilent	12672	634	158	792	20	8	180	112
4	8	0.83	Agilent	12672	634	158	792	20	8	180	113
5	11	0.83	Agilent	12672	634	158	792	20	8	180	115
6	6	0.88	Agilent	12672	576	144	720	22	8	197	116
7	8	0.88	Agilent	12672	576	144	720	22	8	196	117
8	11	0.88	Agilent	12672	576	144	720	22	8	197	119
9	6	0.75	Agilent	12672	792	198	990	16	8	156	108
10	8	0.75	Agilent	12672	792	198	990	16	8	157	110
11	11	0.75	Agilent	12672	792	198	990	16	8	157	112
12	6	0.83	Agilent	12672	634	158	792	20	8	189	117
13	8	0.83	Agilent	12672	634	158	792	20	8	188	118
14	11	0.83	Agilent	12672	634	158	792	20	8	189	120
15	6	0.88	Agilent	12672	634	158	792	20	8	188	111
16	8	0.88	Agilent	12672	634	158	792	20	8	188	112
17	11	0.88	Agilent	12672	634	158	792	20	8	188	114

Supplementary Table S4. Primer sequences for targeting Pool B

File	Forward Primer (5'-3')	Reverse Primer (5'-3')
0	GCTACATGTATACTGCGAGACAGAC	GAGTGATGTGCGACTGCGACTATCG
1	TCTATCTACTCGTGCTCGCTAGCTG	ACATGCGACTAGTGCAGTGCAGACA
2	TGAGATCACAGCTACATAGTGAGAG	TGACTGCACACGACAGTGCTCTATC
3	AGCGTACACGACTGAGCACACTACG	TGCTAGCAGATGCGTGTGAGCGCAT
4	CATCAGCAGTAGAGAGTAGCGCGAT	GCAGACACTGCTAGCGTCGATGATA
5	GAGTCTCTAGCGCTACGAGATATAT	ACAGTCTGCTGATCTCATCAGAGCT
6	CACGAGATCTCAGTGTCGACACGTG	TAGCTGCGTCGAGATCACTATCACT
7	CGCTGCAGTCTATCTCTCTGTACAT	TCTCGCGAGATGTATCTCTACTAGC
8	GTGACTCTGCATATGCTGTCTCGAT	GTGCATCTCACGTGCACACACTCAG
9	ATGAGAGAGAGCGCTCTCTCATGAG	GTACTATGTACATCTATCGCGCTGT
10	GTCGTACTGTGACTCTCACTCGTGC	AGACATGTGTCGACACGTACGCAGA
11	CGCACGCATATCACGATGAGTAGCT	TGAGAGAGCTACTATATGTCTCGCA
12	CGTATGCTCGTATGAGCATAGAGTG	CATCACATCGATACATCTAGACGAC
13	GTGTGAGCTATGCGAGCGACGATCT	TGACGTACAGTGTACGACGTCGACG
14	TATGTACTAGCTAGTCACGCACACA	CGTGAGACTGACGACACAGCAGTGT
15	CTAGACGTGCGAGTATACTACTATG	TCACACGCGTCTGACTGCGTGTCAT
16	TGATCTATGATCGATGTACAGCGCG	AGTGTCAGAGATCGTGCGCGTGAGA
17	TCTCGCTCGAGCACAGAGATAGCGA	GCTCACAGCATCTACAGATACATGT

Supplementary Table S5. Percent of reads decoded correctly (list size 8 for decoding) across values of convolutional code memory (m) and rate (r). These results are for the subpools comprising of a single CRC.

Convolutional code parameter	Memory m = 6	Memory m = 8	Memory m = 11
Rate r = 3/4	59.45%	62.31%	71.30%
Rate r = 5/6	43.42%	55.10%	63.97%
Rate r = 7/8	39.07%	50.02%	53.65%

Supplementary Table S6. Impact of improved basecalling pipeline and improved barcode removal on the percent of reads decoded correctly (list size 8 for decoding). Flappie was used as the basecaller in Chandak et al. (2020), and the data from that study was used for these experiments. Guppy 3.4.5 is a basecaller from Oxford nanopore that is the production version of Flappie.

Convolutional code memory (m)	Convolutional code rate (r)	Flappie	Guppy 3.4.5	Bonito	Bonito + improved barcode removal
8	1/2	68.93%	72.52%	74.20%	87.10%
8	3/4	26.90%	31.87%	34.40%	58.90%
8	5/6	21.67%	26.91%	37.10%	39.60%
11	1/2	62.80%	65.90%	62.90%	88.30%
11	3/4	39.62%	45.13%	58.30%	67.70%
11	5/6	25.91%	33.02%	44.30%	53.40%

Supplementary Table S7. Nanopore decoding results.

Supplementary Table 7A. Basecaller error rate (guppy 4.0.14)

file#	m	r	Unconjugated	Iteration 1	Iteration 2	Iteration 3	Iteration 4	Iteration 5
0	6	0.75	5.36%	5.37%	5.31%	5.54%	5.35%	5.46%
1	8	0.75	5.58%	5.63%	5.60%	5.85%	5.61%	5.82%
2	11	0.75	5.79%	5.87%	5.79%	5.98%	5.95%	5.95%
5	11	0.83	5.65%	5.64%	5.59%	5.85%	5.69%	5.72%
8	11	0.88	5.72%	5.86%	5.76%	5.94%	5.78%	6.02%

Supplementary Table 7B. Coverage analysis (at 5x coverage subsampling): Normalized coverage variance - normalized by the variance for ideal Poisson sampling

file#	m	r	Unconjugated	Iteration 1	Iteration 2	Iteration 3	Iteration 4	Iteration 5
0	6	0.75	1.32	1.39	1.57	1.60	1.72	1.77
1	8	0.75	1.33	1.45	1.59	2.74	15.90	19.38
2	11	0.75	1.47	1.64	1.50	1.49	1.62	1.89
5	11	0.83	1.90	1.86	1.86	1.76	1.79	2.00
8	11	0.88	1.28	1.26	1.55	1.49	1.83	2.01

Supplementary Table 7C. Fraction of oligos with 0 reads (at 5x coverage subsampling)

file#	m	r	Unconjugated	Iteration 1	Iteration 2	Iteration 3	Iteration 4	Iteration 5
0	6	0.75	0.016	0.017	0.016	0.018	0.020	0.022
1	8	0.75	0.012	0.019	0.016	0.031	0.031	0.034
2	11	0.75	0.019	0.022	0.014	0.016	0.018	0.028
5	11	0.83	0.028	0.016	0.037	0.032	0.024	0.066
8	11	0.88	0.006	0.010	0.014	0.019	0.018	0.018

Supplementary Table 7D. Reading cost (success in 10 trials, number of reads used incremented in steps of 250)

file#	m	r	Unconjugated	Iteration 1	Iteration 2	Iteration 3	Iteration 4	Iteration 5
0	6	0.75	3.65	3.65	3.65	3.94	3.94	4.50
1	8	0.75	3.97	3.97	4.54	4.25	4.54	5.39
2	11	0.75	3.17	3.46	3.17	3.17	3.46	3.75
5	11	0.83	3.69	3.69	3.97	3.69	3.40	4.25
8	11	0.88	4.40	4.40	4.70	4.70	4.70	5.58

Supplementary Table 7E. Number of reads for decoding (success in 10 trials, number of reads used incremented in steps of 250)

file#	m	r	Unconjugated	Iteration 1	Iteration 2	Iteration 3	Iteration 4	Iteration 5
0	6	0.75	3250	3250	3250	3500	3500	4000
1	8	0.75	3500	3500	4000	3750	4000	4750
2	11	0.75	2750	3000	2750	2750	3000	3250
5	11	0.83	3250	3250	3500	3250	3000	3750
8	11	0.88	3750	3750	4000	4000	4000	4750

Supplementary Table 7F. Percent correctly decoded

file#	m	r	Unconjugated	Iteration 1	Iteration 2	Iteration 3	Iteration 4	Iteration 5
0	6	0.75	51.08%	52.06%	52.39%	51.05%	51.32%	47.28%
1	8	0.75	50.58%	50.83%	46.93%	47.03%	47.39%	40.09%
2	11	0.75	61.62%	61.16%	63.29%	62.64%	60.46%	56.07%
5	11	0.83	51.82%	52.41%	49.34%	49.46%	51.94%	47.80%
8	11	0.88	37.49%	39.00%	37.82%	36.75%	37.66%	31.82%

Supplementary Table S8. Comparison to other platforms

Writing cost (bases/bit)	Reading cost (bases/bit)	Min Coverage	Technology	Paper	Experimental parameters
0.65	6.8	10.46	Illumina	Erlich and Zielinski (2017)	
0.91	4.55	5	Illumina	Organick et al. (2018)	
0.91	31.39	34.49	Nanopore	Organick et al. (2018)	
0.91	20.02	22	Nanopore	Lopez et al. (2019)	
0.91	2.73	3	Illumina	Chandak et al. (2019)	LDPC w/ 50% redundancy
0.67	3.82	5.7	Illumina	Chandak et al. (2019)	LDPC w/ 10% redundancy
0.93	7.01	7.54	Nanopore	Chandak et al. (2020)	m=11,r=5/6
1.07	6.59	3.56	Nanopore	Chandak et al. (2020)	m=14,r=3/4
1.85	4.42	4.13	Nanopore	Chandak et al. (2020)	m=14,r=1/2
0.99	3.65	3.69	Nanopore	This work	m=6, r=3/4
1.00	3.97	3.98	Nanopore	This work	m=8, r=3/4
1.02	3.17	3.13	Nanopore	This work	m=11, r=3/4
0.90	3.69	4.10	Nanopore	This work	m=11, r=5/6
0.85	4.40	5.21	Nanopore	This work	m=11, r=7/8

Supplementary Table S9. CBA sequences

Subunit	Oligonucleotide #	Barcode sequence	Oligonucleotide sequence (5'-3')
1	1	GGTATATAGTGTCTTCTTGA	AGTGGAGTTCTCCGCATCAAGGTATATAGTGTCTTCTTGATACTGTAGTGCTGCCTTAAT
1	2	GTGCCAAGAACGCACTAGGA	AGTGGAGTTCTCCGCATCAAGTGCCAAGAACGCACTAGGATACTGTAGTGCTGCCTTAAT
1	3	CTGGTCTCTTCGGTCTGGAT	AGTGGAGTTCTCCGCATCAACTGGTCTCTTCGGTCTGGATTACTGTAGTGCTGCCTTAAT
1	4	TCTACTAATCAAGCGTTCGT	AGTGGAGTTCTCCGCATCAATCTACTAATCAAGCGTTCGTTACTGTAGTGCTGCCTTAAT
1	5	CTCTAATGTCCGAGCACACA	AGTGGAGTTCTCCGCATCAACTCTAATGTCCGAGCACACATACTGTAGTGCTGCCTTAAT
1	6	CTCTCCACTGTGACAAGTTG	AGTGGAGTTCTCCGCATCAACTCTCCACTGTGACAAGTTGTACTGTAGTGCTGCCTTAAT
1	7	ATACGACATAACTTCCGCTG	AGTGGAGTTCTCCGCATCAAATACGACATAACTTCCGCTGTACTGTAGTGCTGCCTTAAT
1	8	ACTATAACTTAACGTGAGCG	AGTGGAGTTCTCCGCATCAAACTATAACTTAACGTGAGCGTACTGTAGTGCTGCCTTAAT
2	9	GGACAACGCCTTCTTCTCAA	TACTGTAGTGCTGCCTTAATGGACAACGCCTTCTTCTCAAAGTATTCATATTAAGACGAA
2	10	CAGGTACTGCGTCTATATGG	TACTGTAGTGCTGCCTTAATCAGGTACTGCGTCTATATGGAGTATTCATATTAAGACGAA
2	11	TACATCTGGAATGATCGTGA	TACTGTAGTGCTGCCTTAATTACATCTGGAATGATCGTGAAGTATTCATATTAAGACGAA
2	12	CGAAGGCAATCGACTAATCG	TACTGTAGTGCTGCCTTAATCGAAGGCAATCGACTAATCGAGTATTCATATTAAGACGAA
2	13	ACGTTATCGGTGCCTCGTAC	TACTGTAGTGCTGCCTTAATACGTTATCGGTGCCTCGTACAGTATTCATATTAAGACGAA
2	14	GGCGTTGTATCTTCCAGCAA	TACTGTAGTGCTGCCTTAATGGCGTTGTATCTTCCAGCAAAGTATTCATATTAAGACGAA
2	15	CGCATACGTGCTTACTCGAG	TACTGTAGTGCTGCCTTAATCGCATACGTGCTTACTCGAGAGTATTCATATTAAGACGAA
2	16	CTGCGTTACGCCAACACCAC	TACTGTAGTGCTGCCTTAATCTGCGTTACGCCAACACCACAGTATTCATATTAAGACGAA
3	17	GTCCAGGTAGAGCCTATGAG	AGTATTCATATTAAGACGAAGTCCAGGTAGAGCCTATGAGCAGGCAACCTCGCAACCTCT
3	18	TTGCATACTTCTTGATGCTT	AGTATTCATATTAAGACGAATTGCATACTTCTTGATGCTTCAGGCAACCTCGCAACCTCT
3	19	AGAACTCACTCGCTGATCGG	AGTATTCATATTAAGACGAAAGAACTCACTCGCTGATCGGCAGGCAACCTCGCAACCTCT
3	20	GTGGAAGTTATCGATGCGAA	AGTATTCATATTAAGACGAAGTGGAAGTTATCGATGCGAACAGGCAACCTCGCAACCTCT
3	21	CTCTATGAACTGTAACCGCC	AGTATTCATATTAAGACGAACTCTATGAACTGTAACCGCCCAGGCAACCTCGCAACCTCT
3	22	TTAATTCCATTGAGAGGTTC	AGTATTCATATTAAGACGAATTAATTCCATTGAGAGGTTCCAGGCAACCTCGCAACCTCT
3	23	TAGACCATATACAAGTCTGG	AGTATTCATATTAAGACGAATAGACCATATACAAGTCTGGCAGGCAACCTCGCAACCTCT
3	24	TCCGAAGCTTGGTGACTCTC	AGTATTCATATTAAGACGAATCCGAAGCTTGGTGACTCTCCAGGCAACCTCGCAACCTCT
4	25	GAATACCGAGCGATACAGAA	CAGGCAACCTCGCAACCTCTGAATACCGAGCGATACAGAAATCACACTAGTGAATCGTGA
4	26	AGGTGGACAGTAATAGTATG	CAGGCAACCTCGCAACCTCTAGGTGGACAGTAATAGTATGATCACACTAGTGAATCGTGA
4	27	GCAAGTACGACAGCCTATTC	CAGGCAACCTCGCAACCTCTGCAAGTACGACAGCCTATTCATCACACTAGTGAATCGTGA
4	28	AGAAGCTCACCTCAAGTGAC	CAGGCAACCTCGCAACCTCTAGAAGCTCACCTCAAGTGACATCACACTAGTGAATCGTGA
4	29	TAACTCCAAGGTGCGGTGGC	CAGGCAACCTCGCAACCTCTTAACTCCAAGGTGCGGTGGCATCACACTAGTGAATCGTGA
4	30	ACCAACGGAATGCTTACTGG	CAGGCAACCTCGCAACCTCTACCAACGGAATGCTTACTGGATCACACTAGTGAATCGTGA
4	31	TCCAAGTATCAGTCCTGGTC	CAGGCAACCTCGCAACCTCTTCCAAGTATCAGTCCTGGTCATCACACTAGTGAATCGTGA
4	32	GCTCGATCTGCTAGATCTAG	CAGGCAACCTCGCAACCTCTGCTCGATCTGCTAGATCTAGATCACACTAGTGAATCGTGA
5	33	AGGTTCACCTCTGGATACGC	ATCACACTAGTGAATCGTGAAGGTTCACCTCTGGATACGCAGGATTGCTGATGGATCTGG
5	34	AGACTTCGTGAGCATGCTGG	ATCACACTAGTGAATCGTGAAGACTTCGTGAGCATGCTGGAGGATTGCTGATGGATCTGG
5	35	TCTATCAGCACCTCTACACA	ATCACACTAGTGAATCGTGATCTATCAGCACCTCTACACAAGGATTGCTGATGGATCTGG
5	36	TGAATCTAGGTGGTTAACAG	ATCACACTAGTGAATCGTGATGAATCTAGGTGGTTAACAGAGGATTGCTGATGGATCTGG
5	37	TTGACGTATCTCATGATACC	ATCACACTAGTGAATCGTGATTGACGTATCTCATGATACCAGGATTGCTGATGGATCTGG
5	38	CGCTTCTTCCAGTCAGCATG	ATCACACTAGTGAATCGTGACGCTTCTTCCAGTCAGCATGAGGATTGCTGATGGATCTGG
5	39	CAGACGACCTATGCGAGAAT	ATCACACTAGTGAATCGTGACAGACGACCTATGCGAGAATAGGATTGCTGATGGATCTGG
5	40	TGAGCATTATGCCTCCTCGC	ATCACACTAGTGAATCGTGATGAGCATTATGCCTCCTCGCAGGATTGCTGATGGATCTGG
6	41	ACGACCTAGACTCACAGCCA	AGGATTGCTGATGGATCTGGACGACCTAGACTCACAGCCAATACGTTAGTTCGGCAGTAT
6	42	TCAACGTTGCTTGGAAGTTC	AGGATTGCTGATGGATCTGGTCAACGTTGCTTGGAAGTTCATACGTTAGTTCGGCAGTAT
6	43	AGTTGCTACCATCCTTCATG	AGGATTGCTGATGGATCTGGAGTTGCTACCATCCTTCATGATACGTTAGTTCGGCAGTAT
6	44	TGAACAACCTGGTGGACTGG	AGGATTGCTGATGGATCTGGTGAACAACCTGGTGGACTGGATACGTTAGTTCGGCAGTAT
6	45	GCGTAAGCGGTGATCGACGC	AGGATTGCTGATGGATCTGGGCGTAAGCGGTGATCGACGCATACGTTAGTTCGGCAGTAT
6	46	CTCATCTGATGGCAGTACCG	AGGATTGCTGATGGATCTGGCTCATCTGATGGCAGTACCGATACGTTAGTTCGGCAGTAT
6	47	TACACTGACTGTCAACCACT	AGGATTGCTGATGGATCTGGTACACTGACTGTCAACCACTATACGTTAGTTCGGCAGTAT
6	48	TCTTAAGATCTTACATAGTC	AGGATTGCTGATGGATCTGGTCTTAAGATCTTACATAGTCATACGTTAGTTCGGCAGTAT

Supplementary Table S10. Decoding speed (on a Intel(R) Xeon(R) CPU E5-2630 v4 @ 2.20GHz processor).

			Time per read (in s) on 1 thread		Reading cost	
m	r	Writing cost	L=1	L=8	L=1	L=8
6	3/4	0.990	0.25	2	4.217	3.374
8	3/4	0.998	1	8	4.254	3.687
11	3/4	1.016	8	64	3.174	2.885
6	5/6	0.875	0.25	2	6.353	4.972
8	5/6	0.883	1	8	3.623	3.065
11	5/6	0.898	8	64	3.687	3.120
6	7/8	0.824	0.25	2	5.435	4.291
8	7/8	0.831	1	8	4.328	3.751
11	7/8	0.845	8	64	3.522	3.228
